# Identification of MHC Ligands Through Allele-Guided Isolation Combined With Machine Learning for Improved MHC Assignment Using ARDisplay-I

**DOI:** 10.1016/j.mcpro.2026.101560

**Published:** 2026-03-27

**Authors:** Shima Mecklenbräuker, Piotr Skoczylas, Paweł Biernat, Badeel KH.Q. Zaghla, Bilge Atay, Mai Hossam, Bartłomiej Król-Józaga, Maciej Jasiński, Victor Murcia Pienkowski, Anna Sanecka-Duin, Oliver Popp, Mohamed Haji, Rafał Szatanek, Philipp Mertins, Jan Kaczmarczyk, Ulrich Keller, Agnieszka Blum, Martin G. Klatt

**Affiliations:** 1 Department of Hematology, Oncology and Tumor Immunology, Campus Benjamin Franklin, Charité- University Medicine Berlin, Berlin, Germany; 2 Ardigen S.A., Krakow, Poland; 3 Aix Marseille Univ, INSERM, Marseille Medical Genetics, MMG, Marseille, France; 4 Max-Delbrück-Center for Molecular Medicine and Berlin Institute of Health, Berlin, Germany; 5 German Cancer Research Center (DKFZ) and German Cancer Consortium (DKTK), Heidelberg, Germany; 6 Berlin Institute of Health at Charité–Universitätsmedizin Berlin, Germany; 7 Berlin Institute of Health at Charité–Universitätsmedizin Berlin, BIH Biomedical Innovation Academy, BIH Charité Clinician Scientist Program, Berlin, Germany

**Keywords:** immunopeptidomics, machine learning, multiallelic data, HLA ligand isolation, post-translational modifications

## Abstract

The isolation of major histocompatibility complex (MHC) ligands and subsequent analysis by mass spectrometry is considered the gold standard for defining targets for T cell-based immunotherapies. However, as many targets of high tumor specificity are only presented at low abundance on the cell surface of tumor cells, the efficient isolation of these peptides is crucial for their successful detection. Here, we demonstrate how optimizing the MHC ligand isolation strategy, based on both the presenting MHC alleles and the individual peptide level, enhances the identification of specific MHC ligands. This ideally acknowledges not only the hydrophobicity but also the post-translational modifications of the respective MHC ligands. To further improve the identification and characterization of MHC ligands, we developed an MHC class I ligand prediction algorithm (ARDisplay-I) that outperforms current state-of-the-art tools when benchmarked against competitors such as netMHCpan 4.1, MixMHCpred, or MHCflurry. Implementing these strategies can augment the development of T cell receptor–based therapies by improving the identification of novel immunotherapy targets and enriching the resources available in the computational immunology field through a superior MHC presentation prediction algorithm.

## Highlights

• Different acetonitrile elutions isolate highly distinct HLA ligand populations.
• Isolation strategies influence peptide recovery on the individual (neo)epitope level.
• We successfully benchmarked ARDIsplay-I a new HLA class I binding predictor.

## Graphical Abstract

**Figure:**
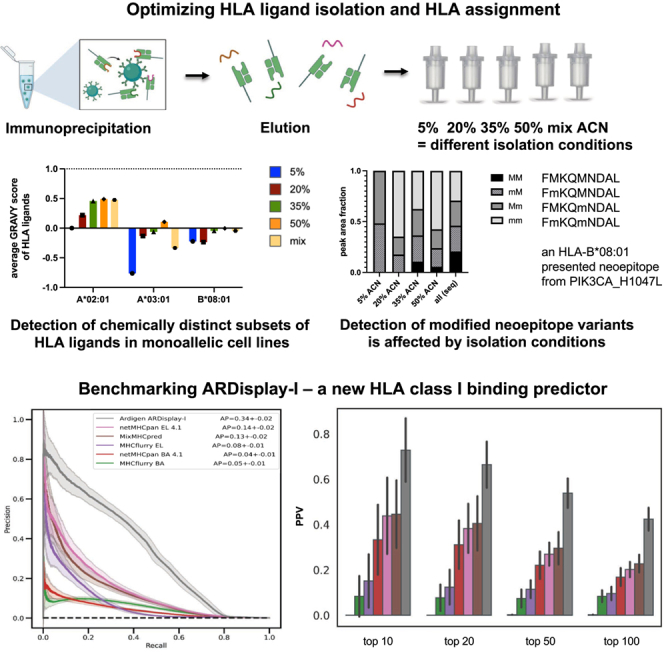

## In Brief

In this study, we demonstrate how the isolation of HLA ligands is dependent on their hydrophobicity, their post-translational modifications, and the HLA allele they are presented on. The effect of different concentrations of acetonitrile for the separation of peptides and HLA complexes were highlighted even on individual peptide level. Additionally, to improve the assignments of these major histocompatibility complex ligands, we developed a machine-learning model to predict the presentation of these major histocompatibility complex ligands, which was successfully benchmarked against widely used algorithms.

## Introduction

Over the last decade, significant improvements have been made in the isolation and identification of major histocompatibility complex (MHC)–bound peptides, now allowing the parallel discovery of thousands of MHC ligands in a single experiment (1, 2, 3, 4). However, different strategies are used for sample preparation, which have a tremendous impact on the isolation of MHC ligands with special biochemical characteristics, such as high hydrophobicity or diverse post-translational modifications (PTMs) (5, 6, 7). Importantly, MHC ligands of high hydrophobicity as well as peptides carrying PTMs (*e.g.,* phosphorylations on serine, threonine, or tyrosine) are more often immunogenic compared to their less hydrophobic or unmodified counterparts (8, 9). Moreover, there has been an increase in the awareness of the importance and underrepresentation of cysteinylated MHC ligands as a potential source of immunogenic peptides (10, 11). Although some studies have used higher concentrations of acetonitrile (ACN) (12) or have investigated the effect of different ACN concentrations on the recovery of hydrophobic peptides (13), very little is known about which MHC allele benefits specifically from using higher concentrations of ACN or if some MHC alleles might benefit more from lower ACN isolation strategies.

Another crucial step in identifying relevant targets for T cell immunotherapy and understanding T cell immune responses is the accurate and precise prediction of potentially antigenic peptides from entire protein sequences, followed by the subsequent assignment of isolated or predicted peptides to their presenting MHC alleles. For this task, different MHC ligand prediction algorithms have been developed (14, 15, 16, 17, 18, 19). Although, peptide-MHC predictors have improved markedly, several benchmarks report elevated false-positive rates (20, 21) and threshold sensitivity for some alleles, and assignment in multiallelic (MA) samples remains ambiguous due to overlapping motifs and deconvolution error (21, 22, 23, 24). Such unclear assignments when multiple MHC alleles compete for the same peptide defines an unmet need to further improve the *in silico* predictions of MHC ligands for T cell-based immunotherapies. However, it should be noted how the MA character of this task increases the complexity of the problem, as each patient has their own particular set of highly polymorphic HLA-I alleles (up to six different types of canonical HLA-I types, i.e., A, B, and C). As of November 2025 (see https://www.ebi.ac.uk/ipd/imgt/hla/), there are over 28,500 known and described canonical HLA-I alleles (8980 HLA-A, 10,725 HLA-B, and 8986 HLA-C alleles), and even though some alleles differ in residues unrelated to the peptide binding, referred to as the binding groove, the number of possible combinations remains enormous. The total number of possible HLA-I sets in a patient, considering that each patient inherits two alleles for each locus (HLA-A, -B, and -C), is approximately 64⋅10^21^ (25). Such complexity and diversity suggest that the problem should be addressed using machine learning approaches based on neural networks. As deep learning approaches require extensive training sets, integrating a variety of data from high-quality sources is a crucial step to achieving high-performance models. We accomplish this in ARDisplay-I by integrating comprehensive MS-eluted ligand (EL) datasets with a customized data-processing pipeline that generates *in silico* large collections of hard negative pHLA pairs (aka decoys). These examples are not displayed on the cell surface, yet closely resemble true binders. Leveraging MS-ELs to train peptide–MHC predictors, as well as protein-matched negative sampling, has been described previously. The improved predictive performance likely reflects a combination of (i) careful curation and integration of training datasets, (ii) a decoy-generation strategy, (iii) the use of a pretrained language model [Bidirectional Encoder Representations from Transformers (BERT)] as the backbone (26), and (iv) training-procedure details. This design, together with class-ratio settings that better reflect MS-derived prevalence and multiple instance learning, yields improved accuracy for MHC-ligand assignment relative to netMHCpan 4.1 (14), MHCflurry 2.0 (15), and MixMHCPred 3.0 (18) as shown herein using an independent, experimentally confirmed set of benchmarking samples.

In parallel, to improve the identification of unmodified, modified, and mutated tumor-specific MHC ligands, we investigated how different isolation strategies based on the biochemical characteristics and the modification status of a peptide can support the isolation and detection of the respective peptide *via* liquid chromatography-tandem mass spectrometry (LC-MS/MS). We corroborated previous results on how the use of higher concentrations of ACN can improve the isolation yield of MHC ligands (6, 13) but can lead to a relative decrease in the isolation efficacy for other MHC alleles. We compared our results for improved MHC ligand isolation using higher concentrations of ACN for specific MHC alleles to the average hydrophobicity of peptides isolated from monoallelic cell lines corresponding to the investigated MHC alleles (12, 27). We observed a highly significant correlation between the results from monoallelic cell lines and our experiments, suggesting that data from monoallelic cell lines inform us which MHC alleles might benefit the most from isolation strategies with high ACN conditions. Additionally, we observed that phosphorylated MHC ligands exhibit improved isolation for lower ACN conditions, aligning with the low hydrophobicity of this modification. Next, to validate our findings, we analyzed the effectiveness of different isolation modes for an exemplifying set of three monoallelic cell lines (HLA-A∗02:01, HLA-A∗03:01, HLA-B∗08:01) that were generated through transfection of COS-7 monkey fibroblasts. Moreover, we demonstrated how different ACN concentrations strongly affect the isolation efficiency of a PIK3CA-derived neoepitope and other peptides, illustrating effects not only on the MHC allele but also on the individual MHC ligand level.

Overall, this study demonstrated that an optimized isolation strategy enhances the isolation of MHC-bound peptides when hydrophobicity and modification status of the peptides are considered. Additionally, we developed a MHC ligand prediction algorithm ARDisplay-I, which surpasses leading tools on an independent dataset of experimentally obtained samples and will support not only the definition of new targets for T cell immunotherapies but might also help to improve, for example, predictors of neoantigen fitness (28), off-target immunotoxicity (29, 30), and response to immunotherapies, which all rely on MHC ligand prediction algorithms (25, 31).

## Experimental Procedures

### Experimental Design and Statistical Rationale

LP-1, JJN3, and Nalm-6 cells were chosen based on their MHC typing and high MHC presentation levels. These cell lines express 16 distinct allotypes. At least one of these alleles is found in more than 92% of the global population, as determined by the IEDB population coverage tool (32) (Supplemental Fig. S1). We performed the main experiments in biological quadruplicates, which allows statistical comparisons between the different ACN conditions. For the analysis of modified peptides, we merged data from three cell lines, which enables comparisons between 12 biological replicates. Validation experiments were performed once in COS-7-transfected monoallelic cell lines and T2 cells.

### Cell Lines

LP-1, JJN3, and Nalm-6 cells were a gift from the Krönke Lab at Charité and maintained at Charité-University Medicine Berlin. The transporter associated with antigen processing (TAP)-deficient T2 cell line was a kind gift from Professor Ochsenreither, Berlin, Germany. The monkey fibroblast COS-7 cell line was acquired and authenticated through the Leibniz Institute DSMZ-German Collection of Microorganisms and Cell Cultures GmbH (DSMZ accession number ACC 60). MHC typings were retrieved from published data or the TRON cell line repository (33). All cell lines were maintained in RPMI media and supplemented with 10% FBS and 2 mM glutamine and regularly checked for *mycoplasma* contamination.

### Synthetic Peptides and T2 Peptide Pulsing

HLA-A∗02-restricted peptides ALNEQIARL, ALNEKLVNL, and MLANDIARL, as well as peptides containing stable isotope-labeled amino acids (ALNEQIAR(+10 Da)L, ALNEK(+8 Da)LVNL, MLANDIAR(+10 Da)L) were synthesized by Biosynth (Flevoland, Nederland). All peptides were synthesized with a purity of more than 90% and verified by mass spectrometry analysis. The peptides were dissolved in DMSO at a concentration of 1 mg/ml and stored at −80 °C until required. For peptide pulsing, T2 cells were seeded at a density of 1 × 10^5^ cells/well in the serum-free RPMI 1640 and then incubated with 3 ng/ml β2-microglobulin (Sigma-Aldrich, Cat# SAE0112) and the above-mentioned peptides at a final concentration of 1 μg/ml at 37 °C for 18 h.

### Immunopurification of MHC Class I Ligands

For immunopurification, suspension cells were harvested by direct resuspension, and adherent cell lines were incubated for 15 min with CellStripper solution (Corning, Cat# 25056CI). Harvested cells were pelleted and washed three times in ice-cold sterile PBS (Gibco). For experiments with LP-1, JJN3, and Nalm-6 cell lines, 150 × 10^6^ cells were used in quadruplicate. For T2 cells, cells were lysed in 7.5 ml of 1% CHAPS (Sigma-Aldrich, Cat# C3023) dissolved in PBS and supplemented with protease inhibitors (cOmplete, Cat# 11836145001). Cell lysis was performed for 1 h at 4 °C, lysates were spun down for 1 h at 20,000*g* at 4 °C, and supernatant fluids were isolated. Affinity columns were prepared as follows: 40 mg of cyanogen bromide-activated-Sepharose 4B (Sigma-Aldrich, Cat# C9142) were activated with 1 mM hydrochloric acid (Sigma-Aldrich, Cat# 320331) for 30 min. Subsequently, 1 mg of W6/32 antibody (BioXCell, Cat #BE0079) was coupled to Sepharose in the presence of binding buffer (150 mM sodium chloride, 50 mM sodium bicarbonate, pH 8.3; sodium chloride: Sigma-Aldrich, Cat# S9888, sodium bicarbonate: Sigma-Aldrich, Cat#S6014) for at least 2 h at room temperature. Sepharose was blocked for 1 h with glycine (Sigma-Aldrich, Cat# 410225) and washed three times with PBS.

Supernatants of cell lysates were run over the columns through peristaltic pumps at a flow rate of 1 ml/min overnight in a cold room. Affinity columns were washed with PBS for 30 min, water for 30 min, then run dry, and MHC complexes were subsequently eluted five times with 200 μl 1% trifluoracetic acid (TFA, Sigma/Aldrich, Cat# 02031). The TFA eluates were pooled and then split into as many portions as settings were investigated (either three portions with 30%, 40%, and 50% ACN settings and five portions with 5%, 20%, 35%, 50%, and “mix” settings).

For the separation of MHC ligands and their MHC complexes, C18 columns (Sep-Pak C18 1 cc Vac Cartridge, 50 mg Sorbent per Cartridge, 37–55 μm Particle Size, Waters, Cat# WAT054955) were prewashed with 80% ACN (Sigma-Aldrich, Cat# 34998) in 0.1% TFA and equilibrated with two washes of 0.1% TFA. Samples were loaded, washed again with 0.1% TFA, and eluted in 400 μl of either 30%, 40%, or 50% ACN in 0.1%TFA for BV173, AML14, and JMN cells or for LP-1, JJN3, and Nalm-6 cells with 4 × 200 ul of either 5%, 20%, 35%, and 50% ACN in 0.1%TFA or 200 ul each of 5%, 20%, 35%, and 50% ACN in 0.1%TFA for the “mix” setting. The sample volume was reduced by vacuum centrifugation for mass spectrometry analysis. For quantitative experiments, 30 pmol of heavy isotope-labeled peptides were added to each sample before mass spectrometry acquisition.

### Plasmids and Transfections

Gene sequences for driver genes and MHC alleles utilized in our research were synthesized and inserted into the pcDNA3.1+ vector by GenScript. We generated constructs encoding full-length driver genes and the MHC alleles of interest. To prepare the RNA for *in vitro* transcription, we first linearized the plasmid vectors with the Xbal restriction enzyme (New England Biolabs). The linearized plasmids were then purified using the QIAquick PCR Purification Kit (Qiagen) as per the manufacturer’s guidelines. In the next step, we synthesized the *in vitro* RNA from the purified linearized plasmids using the HiScribe T7 High Yield RNA Synthesis Kit (New England Biolabs), following the protocol provided by the manufacturer. COS-7 cells were co-electroporated with 100 μg/ml of mRNA encoding an individual MHC allele along with the driver protein using the Neon Transfection system (10 μl tip, 1050 V/30 ms/2 pulses).

### Solid Phase Extractions

In-house C18 mini columns were prepared as follows: for solid phase extractions of one sample, two small disks of C18 material (1 mm in diameter) were punched out from CDS Empore C18 disks (Fisher Scientific, Cat# 13-110-018) and transferred to the bottom of a 200 μl Axygen pipette tip (Fisher Scientific, Cat# 12639535). Columns were washed once with 100 μl 80%ACN/0.1%TFA and equilibrated with three times 100 μl 1%TFA. All fluids were run through the column by centrifugation in mini tabletop centrifuges, and eluates were collected in Eppendorf tubes. Then, the dried samples were resuspended in 100 μl 1% TFA and loaded onto the columns, washed twice with 100 μl of 1% TFA, dried, and eluted with 50 μl 80%ACN/0.1% TFA. Again, the sample volume was reduced by vacuum centrifugation.

### LC-MS/MS Analysis of MHC Ligands

Samples were analyzed by LC-MS/MS (Orbitrap Exploris, Thermo Fisher) 480 mass spectrometers (Thermo Fisher Scientific). A 44 min gradient was applied using an EASY-nLC 1200 system (Thermo Fisher Scientific) with an in-house packed column (C18-AQ 1.9 μm beads; Dr Maisch Reprosil-Pur 120). MS1 resolution was set to 120′000, a MIPS peptide filter with relaxed restrictions was applied, the minimum intensity threshold was specified to 50′000, dynamic exclusion occurred for 20 s, and charge states 1 to 5+ were allowed as precursors.

### Mass Spectrometry Data Processing

Mass spectrometry data were processed using Byonic software (34) (version 4.5.2, Protein Metrics) and PEAKS software (35) (version 11, Bioinformatics Solutions Inc.) through a custom-built computer server. Mass accuracy for MS1 was set to 10 ppm and 20 ppm for MS2, respectively. Digestion specificity was defined as unspecific, and only precursors with charges 1, 2, 3, and up to 2 kDa were allowed. Protein FDR was disabled to allow a complete assessment of potential peptide identifications. Oxidation of methionine, cysteine, histidine, lysine, proline, and tryptophan, phosphorylation of serine, threonine, and tyrosine, acetylation of lysine and the protein N terminus, methylation of cysteine, aspartate, glutamate, histidine, leucine, asparagine, glutamine and arginine, dimethylation of lysine and arginine, as well as cysteinylation of cysteine were set as variable modifications for all samples. For PEAKS software analyses, only oxidation of methionine, phosphorylation of serine, threonine, and tyrosine, as well as cysteinylation of cysteine, were set as variable modifications for all samples. Samples were searched against the UniProt Human Reviewed Database with common contaminants added (downloaded October 10, 2022, 43,277 entries searched). For COS-7 cells, a UniProt database for *Chlorocebus aethiops*, including unreviewed entries, was searched (downloaded September 24, 2024, 52,388 entries searched). Peptides were selected with a minimal log prob value of 1.3, indicating *p*-values for PSMs of <0.05, and duplicates were removed so that no multiple PSMs with the same peptide sequence were counted twice. For calculating molecules per cell in quantitative immunopeptidome experiments, relative peak areas for heavy and light-labeled peptides were determined through Skyline software (36) (version 24.1, MacCoss Lab Software).

### Assignment of Peptide Sequences to MHC Alleles

To assign peptides that passed the MS quality filters described above to their MHC alleles, which they are most likely to bind, we used the netMHCpan 4.1 algorithm with default settings (14) and the ARDisplay-I presentation model. No binding affinity (BA) predictions were enabled.

**Fig. 1:**
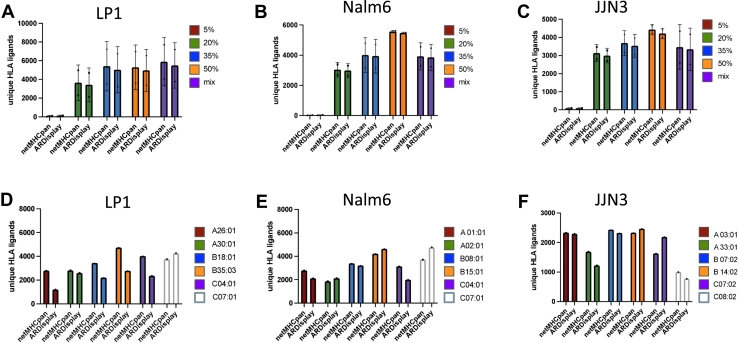
**Comparison of netMHCpan with ARDisplay-I for assigning MHC ligands to MHC alleles.***A–C*, *D–F*, absolute numbers of unique MHC ligands between different ACN elution conditions and (*D–F*) for different MHC alleles expressed in LP-1, Nalm6 and JJN3 cells. The following thresholds were used: Ardigen ARDisplay (≥50%) and netMHCpan (<2.0% rank). ACN, acetonitrile; MHC, major histocompatibility complex.

**Fig. 2:**
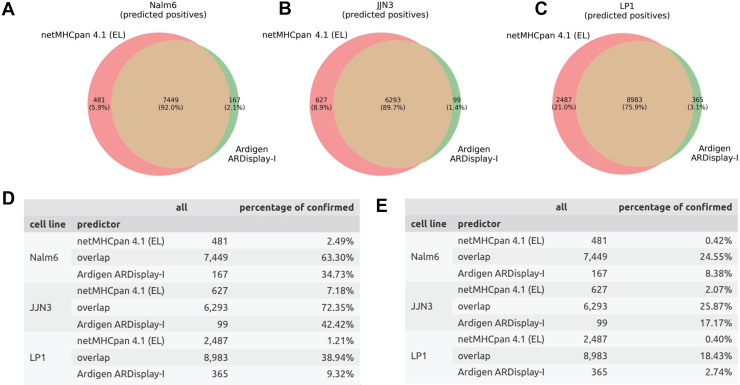
**Comparison of netMHCpan with ARDisplay-I for assigning MHC ligands to MHC alleles.***A–C*, *D–E*, Venn diagrams with numbers of pMHC pairs predicted by the respective algorithm, or both, as presented on the cell surface. Tables showing the numbers of predicted observations, along with the numbers and percentages of those that were confirmed using single-allelic data sets in the two described benchmarking scenarios (see the main text for details). The following thresholds were used: Ardigen ARDisplay (≥50%) and netMHCpan (<2.0% rank). MHC, major histocompatibility complex.

**Fig. 3:**
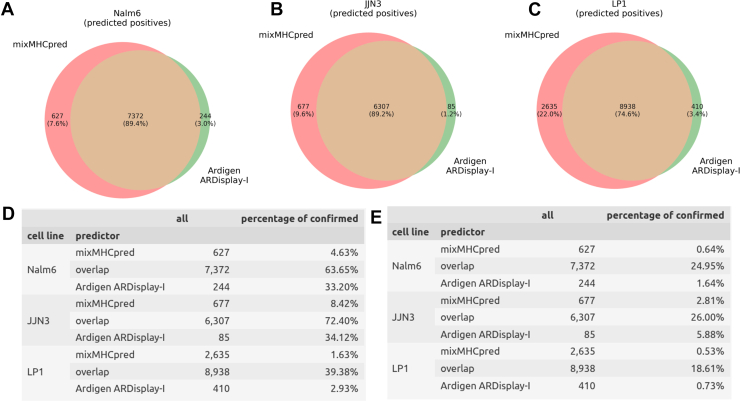
**Comparison of mixMHCpred with ARDisplay-I for assigning MHC ligands to MHC alleles.***A–C*, *D–E*, Venn diagrams with numbers of pMHC pairs predicted by the respective algorithm, or both, as presented on the cell surface (*D–E*). Tables showing the numbers of predicted observations, along with the numbers and percentages of those that were confirmed using single-allelic data sets in the two described benchmarking scenarios (see the main text for details). The following thresholds were used: Ardigen ARDisplay (≥50%) and MixMHCpred (80% quantile) to obtain similar numbers of overlapping peptides as in the previous comparison (see Fig. 2). MHC, major histocompatibility complex.

**Fig. 4:**
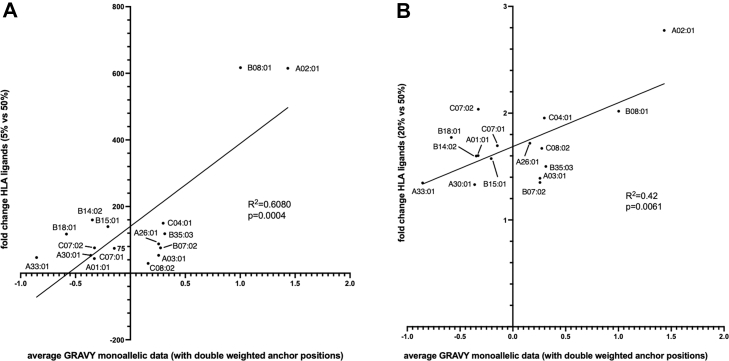
**Correlation of the increase in MHC ligand yields per MHC allele and the average hydrophobicity of peptides eluted from monoallelic cell lines.***A,* average hydrophobicity of MHC ligands was calculated for peptides reported by Sarkizova *et al*. (27) and Abelin *et al*. (12) from monoallelic cell lines through GRAVY scores. GRAVY scores of amino acids at anchor positions were counted twice. For better comparability, only 9mers were used. Data were correlated with fold changes of MHC ligands from experimental data (5% and 50% ACN conditions). Peptides isolated in these conditions with a predicted %rank of <0.05 were considered binders to reduce the risk of ambiguous HLA assignments. *B,* same comparison as in (*A*), but comparing 20% and 50% ACN conditions. For HLA assignments in the experimental setting a higher threshold of Ardigen ARDisplay ( ≥ 95%) was used to increase the probability for monoallelic assignments. ACN, acetonitrile; MHC, major histocompatibility complex.

To avoid favoring, we adopted method-native, widely used operating points: for netMHCpan (EL), we used %Rank ≤2 for binders; for ARDisplay-I, we used ≥0.5 for all comparisons in Fig. 1, Fig. 2, Fig. 3. Only for Figure 4, a stricter threshold of ≥0.95 was used to increase the chance for monoallelic assignments that were crucial for Figure 4. Still, our primary conclusions regarding overall performance of ARDisplay-I rely on threshold-free metrics (precision-recall curves, area under the precision-recall curve, PPV@k) in the MA setting.

### Software and Statistics

All graphs except the Venn diagrams were drawn with GraphPad Prism 7. For statistics, built-in analyses from GraphPad Prism were used. One-way ANOVA tests with Friedmann’s multiple comparison tests were used for comparing GRAVY scores. Venn diagrams were prepared using the Venn diagrams tool by the University of Gent. (https://bioinformatics.psb.ugent.be/webtools/Venn/).

### Training of ARDisplay-I: HLA Ligand Presentation Model

A curated dataset containing peptides presented by class I HLAs on the surface of host cells was extracted from more than 20 publicly available datasets from selected publications. MS experiments provided by each group experimentally confirmed the presence of each HLA ligand. All peptides were of human origin and were presented on the surface of either monoallelic human cell lines or MA data obtained from healthy donors or cancer patients. Synthetic negative data (*i.e.,* nonpresented peptides, decoys) were prepared based on the human proteome (GRCh38, release 98). Only peptides containing 8 to 11 naturally occurring amino acids were taken into consideration. To enhance model robustness, a set of protein-matched candidate negatives was limited to hard negatives, *i.e.,* peptides that are not displayed yet are difficult to distinguish from true ligands. We achieved this by retaining only candidates with predicted BA ≤2000 nM to the corresponding HLA (MHCflurry BA). We additionally removed any candidates overlapping known positives. The resulting negatives are difficult to distinguish from true ligands and reduce false-positive inflation during training.

The current version of ARDisplay-I is a classifier combining a pragmatic treatment of MA data (*i.e.,* by extracting all possible pHLA pairs, generating a model prediction for each, applying filtering based on BA predictions from MHCflurry, and re-aggregating the results) with protein language model embeddings from BERT (37)/tasks assessing protein embedding (TAPE) (38) and an attention-based multiple instance learning aggregator that operates over candidate HLA instances. The BERT is an NLP family of models based on the transformer architecture. Such a model was applied to selected tasks involving protein biology—see TAPE. One of the TAPE models, pretrained for protein representation learning, was used as an initial step in the training of ARDisplay-I. Transformer encoders (including BERT-style protein language models) have been explored for MHC binding/presentation (26). These studies show that pretrained sequence encoders can provide strong representations, while downstream performance depends heavily on training data, negative sampling, and data balancing strategies. Our design follows this line: we adopt a pretrained encoder and focus on dataset design and decoy strategy to reduce false-positive inflation in MA settings.

ARDisplay-I accepts peptides of 8 to 11 AA without truncation. Each peptide is tokenized and passed through the pretrained ProteinBERT encoder, which includes positional embeddings. Sequences are right padded only within each batch to the batch-max length using the [PAD] token, and a standard attention mask ensures padding tokens do not contribute to the pooled representation. We then use the pooled [CLS] embedding as the peptide vector.

Our model outputs the presentation probability of a given pHLA pair. Moreover, ARDisplay-I can be used to predict whether a peptide will be displayed *via* any of the patients’ HLA molecules. To ensure that ARDisplay-I can make predictions on a vast set of known HLA alleles, we used the pseudosequences provided in the PUFFIN model (39) to encode the HLA types. Such pseudosequences describe the amino acids from the HLA that form a binding groove and have the potential to interact with the peptide that they bind.

We encoded peptides and HLA pseudosequences using the TAPE *ProteinBertModel* initialized from the *bert-base* checkpoint and took the pooled CLS token as the sequence embedding. Concatenated peptide/HLA embeddings were fed to an attention-based multiple instance learning aggregator that operates over candidate HLA instances per peptide (classifier_hidden_size: 128, mil_hidden_size: 128). A lightweight MLP with a sigmoid transformation maps predictions to probability. Training used label smoothing = 0.1 (to optimize BCE-with-logits loss), learning rate = 1 × 10^−4^, weight decay = 1 × 10^−4^, batch size 512 with gradient accumulation = 2, and early stopping on validation loss with patience = 10.

ARDisplay-I was implemented in Python 3.9 using the PyTorch v.1.13.1 library, enabling performing fast operations on tensors and neural networks with GPU acceleration. Additionally, we have used PyTorch Lightning v.2.0.0 for the maintenance and training of our model. GPU-based computations were performed on a machine equipped with NVIDIA Ampere A100 GPU cards featuring CUDA 8.6 architecture, 640 Tensor Cores, 6912 CUDA Cores, and 40 GB HBM2 GPU Memory, and using cudnn 8.5.0 and cudatoolkit 11.7.99. Standard Python libraries for data analysis and machine learning were utilized (among others, scikit-learn, pandas, numpy, matplotlib, and seaborn).

### Metrics Used for Benchmarking

The precision-recall curve is a standard curve used to evaluate a binary classification model. It is especially beneficial for very imbalanced data. It visualizes a trade-off between precision (the proportion of positive predicted samples that are true positives) and recall (the fraction of all positives that the model detects). Average precision (AP) is the average of precision values at different recall levels, *i.e.,* a proxy to the area under the precision-recall curve.

Precision (also positive predictive value, PPV) is a metric that describes the percentage of positively classified cases in binary classification that are true positives. In the case of presentation models, PPV provides information on the fraction of the pHLA pairs, labeled by the model as presented, that were indeed presented in the experimental setup. Typically, PPV estimation requires defining a threshold above which an observation is classified as positive. For example, the PPV for the top 100 describes how many true positive observations can be expected in the lab by testing the 100 pHLA pairs with the highest ranks as determined by the model predictions.

### Model Availability and Usage

The model used in this study is available *via* Hugging Face (see https://huggingface.co/) for noncommercial academic use. It was developed at Ardigen as part of the Immunology platform, and a free version can be accessed through Hugging Face (see License for details, https://huggingface.co/ardigen/ardisplay-i/blob/main/LICENSE.md). For commercial use or access to Pro versions, please contact ardisplay@ardigen.com.

The model can be found and used online *via* Hugging Face servers (model official repository: https://huggingface.co/ardigen/ardisplay-i) or downloaded locally and used interactively *via* Python or as a CLI tool in bioinformatics pipelines.

In most applications, additional postprocessing and filtering may be required. When working with nonstandard data (*e.g.,* neoepitopes, viral peptides, alternative splicing products, and dark antigens), further domain knowledge or fine-tuning may be necessary.

## Results

### Higher Concentrations of Acetonitrile Favor MHC Ligand Isolations, but Different Concentrations Favor Distinct MHC Ligand Subgroups

**Fig. 5:**
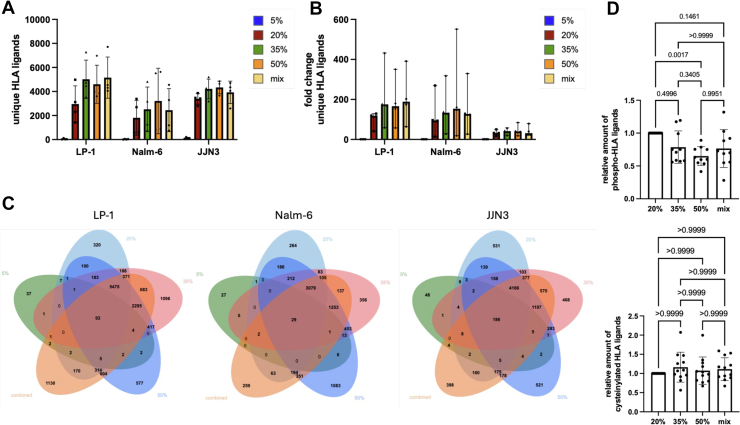
**Effect of various acetonitrile elution conditions on the isolation efficiency of modified and unmodified MHC ligands.***A,* unique MHC ligands isolated from the same pool of peptide:MHC using various concentrations of acetonitrile (ACN) in JJN3, LP-1, and Nalm-6 cells. *B,* relative changes for the yields of unique MHC ligands between different ACN elution conditions in JJN3, LP-1, and Nalm-6 cells. *C,* Venn diagrams illustrating the overlap of unique MHC ligands in various ACN conditions for LP-1, Nalm-6, and JJN3 cells. *D,* the relative amount of modified MHC ligands isolated from LP-1, Nalm-6, and JJN3 cells in various ACN conditions—phosphorylated (*top*) and cysteinylated (*bottom*). Data were normalized to samples with 20% ACN. Data are shown for biological quadruplicates. MHC, major histocompatibility complex.

In a previous study, we demonstrated that the most influential step in our MHC ligand isolation protocol is the separation of peptides and MHC complexes after loading them onto C18 cartridges (6). This separation is achieved by selectively eluting the MHC ligands while leaving the complexes on the C18 material, using ACN. Therefore, we first sought to investigate how using a broader range of ACN concentrations influences the effectiveness of the peptide elution. We hypothesized that highly hydrophobic peptides could benefit from high ACN concentrations, whereas very hydrophilic peptides could be better isolated with very low ACN concentrations. For our experimental conditions, we chose ACN concentrations of 5%, 20%, 35%, and 50%. Sequential use of all four concentrations was defined as “mix”. Three different cell lines, including two multiple myeloma lines (JJN3 and LP-1) and the lymphoblastic leukemia cell line (Nalm-6), were selected for their broad MHC allele repertoires. Of note, from the global population, over 92% of individuals carry at least one of the investigated allotypes (supplemental Fig. S1). MHC complexes of all cell lines were isolated using the HLA-A–, HLA-B–, and HLA-C–specific W6/32 antibodies, and MHC complexes were eluted with TFA. These eluates were then split into five equal parts and loaded onto C18 cartridges for the five experimental steps. For all ACN concentrations, we observed a clear increase in the number of detectable MHC ligands over all three cell lines and across their four biological replicates (Fig. 5*A*, supplemental Data 1, *A*–*C*). However, the 5% ACN condition was by far the least effective condition as it could result on average in an up to 200-fold less effective isolation compared to the elution with 50% ACN (Fig. 5*B*). With 20% ACN, a sufficient isolation of peptides was achieved although MHC ligand yields could still be further increased when using 50% ACN for elution (Fig. 5*A*). Interestingly, although expected to result in the highest peptide yields, the subsequent use of all different ACN conditions did not always result in the highest numbers of isolated MHC ligands (Fig. 5*B*). Still, the subgroups of isolated MHC ligands were found to be highly variable between the five different isolation strategies with 10 to 35% of total peptides identified only in one distinct ACN setting (Fig. 5*C*).

We then investigated how the different concentrations of ACN affected the recovery of modified MHC ligands. As hypothesized, the biochemical characteristics of the PTMs contributed to the effectiveness of MHC ligand isolation. The addition of a very hydrophilic phosphate group led to a significantly better isolation of modified MHC ligands in the 20% ACN setting over the 50% ACN setting compared to the isolation of unmodified MHC ligands over all three cell lines (Fig. 5*D*, *top*). In contrast, for all the other modifications tested (cysteinylation, oxidization, acetylation, methylation, and dimethylation), no significant differences could be detected (Fig. 5*D* *bottom*, supplemental Fig. S2). For larger modifications like ubiquitinations, sumoylations, or FAT-modification, only minimal subsets of peptides were identified, which did not allow a systematic analysis. Small alterations, such as deamidations, were not tested as we did not expect significant changes due to the small mass shift and little biological consequences for T cell recognition. Similar to the results for unmodified peptides, regardless of the type of modification, MHC ligands specific for each ACN concentration were observed. Interestingly, the observation that most shared nonmodified MHC ligands could be identified in the intersection of 20%, 35%, 50%, and sequential elution conditions was maintained in oxidized and phosphorylated MHC ligands, but methylated, demethylated, and acetylated MHC ligands showed a very unique distribution with most MHC ligands being detected in the unique section of every ACN condition. This suggests that the latter modification might be more susceptible to different ACN-based elution strategies (supplemental Fig. S3).

Overall, we demonstrated that higher ACN concentrations correlate with higher numbers of identified MHC ligands, although a minimum of 20% seems necessary for sufficient isolation. Additionally, we demonstrated how the amount of ACN used favors the isolation of distinct MHC ligand subgroups and affects the isolation of post-translationally modified MHC ligands in dependence on their biochemical characteristics.

### A Wide Range of Acetonitrile Concentrations Identifies MHC Alleles That Benefit From the Use of High or Low Acetonitrile Concentrations

**Fig. 6:**
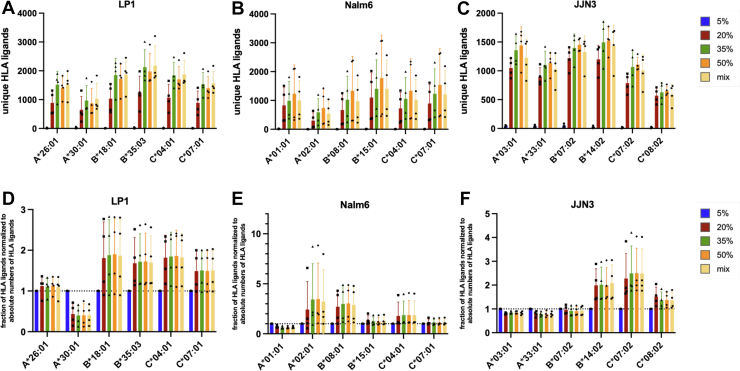
**Absolute and relative changes in unique MHC ligands by MHC allele.***A–C,* relative changes for the yields of unique MHC ligands between different ACN elution conditions for different HLA alleles expressed in JJN3 (*A*), LP-1 (*B*), and Nalm-6 (*C*) cells. Results are normalized to a 5% ACN setting and relative to the total number of MHC ligands per condition. *D–F,* MHC ligands were assigned to respective alleles by netMHCpan using a 2% rank cutoff. Absolute numbers of MHC ligands assigned to a specific MHC allele. All experiments were performed in biological quadruplicates. ACN, acetonitrile; MHC, major histocompatibility complex.

As the effect of ACN on MHC ligand isolation depends on the corresponding MHC allele and the resulting anchor amino acids (6), we next wanted to broaden the knowledge about MHC alleles that benefit from extended or limited use of ACN during the isolation process. For this purpose, we analyzed 16 different MHC class I alleles expressed on the JJN3, LP-1, and Nalm-6 cells and investigated how the isolation of MHC-specific ligands changed with varying ACN concentrations. First, all identified MHC ligands were assigned to the MHC allele/s, they are predicted to be presented by, as defined by netMHCpan 4.1 (Fig. 6, *A*–*C*), and we then calculated the fractions of MHC ligands relative to the total number of MHC ligands per sample. These results were normalized to the 5% ACN samples, which, although low in total MHC ligand yields, provided insights about MHC alleles that favor peptides with very low hydrophobicity. Finally, these fractions were compared to the increase in the number of MHC ligands over different ACN conditions (Fig. 6, *D*–*F*). Using this analysis, we were able to describe MHC alleles A∗02:01, B∗08:01, B∗14:02, B∗18:01, B∗35:03, C∗04:01, C∗07:01, and C∗07:02 as those that benefit the most from the use of higher ACN concentrations, with the strongest increases for A∗02:01 and B∗08:01 in line with previous results (6). In contrast, A∗01:01, A∗03:01, A∗33:01, and most profoundly A∗30:01 demonstrated relative decreases in MHC ligand isolations (Fig. 6, *D*–*E*). It is important to note that despite the relative decreases observed for some alleles, all MHC alleles showed an overall increase in the absolute number of isolated MHC ligands when higher ACN concentrations were used, as shown in (Fig. 6, *A*–*C*).

Furthermore, to ensure that the observed effects between MHC alleles are significant and not related to variation between replicates, we performed Tukey’s multiple comparison test. Although this test cannot determine which MHC alleles would benefit from the use of more or less ACN, it highlights the strong differences between alleles. We summarized these data in a heatmap based on the obtained *p*-values for comparing the different MHC alleles (supplemental Fig. S4). Overall, these data suggest that the preference for peptide isolation is based on biochemical characteristics rather than the availability of peptides on a specific allele.

To further corroborate these results, we repeated our analyses for unmodified and some of the modified peptides (oxidation, cysteinylation, and phosphorylation) using the PEAKS software, as this package is specifically for immunopeptidome analyses. Of note, the number of absolute and relative recovered MHC ligands between different ACN conditions (supplemental. Fig. S5, *A* and *B*), their distribution between MHC alleles (supplemental Fig. S5, *C*–*E*) and the recovery of modified MHC ligands (supplemental Fig. S6, *A*–*C*) were highly comparable.

To investigate more systematically whether MHC ligands of a particular allele would benefit from the use of high concentrations of ACN, we then compared the increase of MHC ligands per allele with the average hydrophobicity of peptides eluted from monoallelic cell lines. Of note, when hydrophobicity scores were calculated for monoallelic data *via* the GRAVY (40) score calculator, we doubled the values for amino acids at anchor positions, as we had previously demonstrated the strong dependence of the MHC ligand isolation from the biochemical characteristics of these amino acids. Additionally, when the increase of the average of hydrophobicity of MHC ligands per allele was calculated, we used the results from the 5% ACN and 50% ACN conditions (and from the 20% *versus* 50% ACN conditions as comparison) and MHC ligands meeting more stringent prediction criteria (0.05 %Rank score as defined by netMHCpan 4.1) to focus mostly on peptides that are uniquely assigned to a single MHC allele rather than multiple alleles, to decrease the risk of the inappropriate match, and to enable comparability with monoallelic data. A clear positive and highly significant correlation (R^2^ = 0.6080, *p* = 0.0004) was found for the increase of MHC ligands from 5% to 50% conditions with the average hydrophobicity of peptides isolated from monoallelic cell lines (Fig. 4*A*). For the comparison of the 20% and 50% conditions, we still observed a strong and significant positive correlation (R^2^ = 0.42, *p* = 0.0061). Still, a comparison between 5% and 50% ACN might be more suitable to underline how different MHC alleles react to increased concentrations of ACN during the MHC ligand isolation process.

Overall, this correlation might suggest which MHC alleles could benefit from more stringent ACN isolation strategies, although the MA character of the used cell lines might bias the observed results.

### Monoallelic and TAP-Deficient Cell Lines Elucidate the Effects of Different Acetonitrile Isolation Strategies

**Fig. 7:**
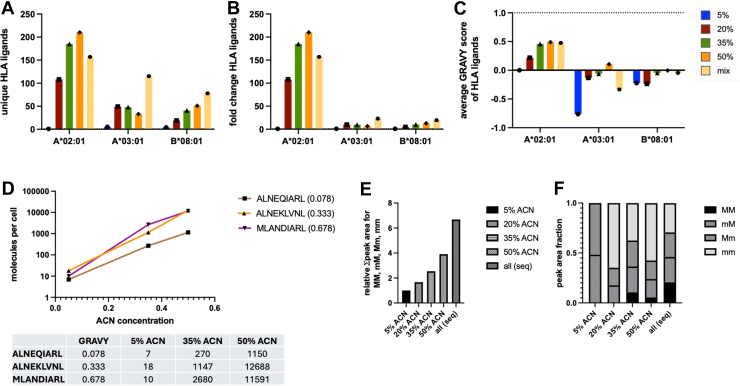
**Confirmation of ACN-dependent MHC ligand isolation in monoallelic cell lines.** COS-7 cells were transfected with either HLA-A∗02:01, HLA-A∗03:01, or HLA-B∗08:01 to create monoallelic cell lines. MHC ligands were isolated from these cell lines using ACN concentrations of 5%, 20%, 35%, 50%, and a sequential elution with all these conditions. *A–C,* absolute number of MHC ligands isolated (*A*), relative number of MHC ligands isolated and normalized to 5% ACN (*B*), GRAVY score of the average hydrophobicity of MHC ligands isolated from the different ACN conditions (*C*). *D,* quantitative immunopeptidomics analysis estimating the number of presented molecules per cell from T2 cells pulsed with 1 μg/ml of three different peptides and isolated with 5%, 35%, and 50% ACN. *E–F,* peak areas from the public neoepitope FMKQMNDAHL presented on COS-7 cells cotransfected with B∗08:01 and PIK3CA H1047L. The neoepitope presentation was assessed in four different states depending on the oxidation state of the two methionines present (M = nonoxidized methionine and m = oxidized methionine) in the neoepitope called MM, mM, Mm, and mm. *E,* combined peak areas for all four peptide variants in different ACN conditions. *F,* peak area distribution of the four peptide variants in the different ACN conditions. ACN, acetonitrile; MHC, major histocompatibility complex.

In the following step, we wanted to validate the MHC allele-specific effects observed in the MA setting in monoallelic cell lines. As stable-transduced monoallelic cell lines were not available in our lab and based on our expertise from previous studies (41), we transfected COS-7 cells, an MHC low monkey fibroblast cell line, *via* electroporation with mRNA encoding HLA-A∗02:01, HLA-A∗03:01, and HLA-B∗08:01. We selected these alleles because they had demonstrated strong effects in our MA experiments. After harvesting and lysing these cell lines, we performed immunoprecipitation of MHC complexes and eluted peptides with TFA. Next, we split the eluate into five fractions as we did for the MA cell lines and loaded the eluates onto five different C18 columns. After elution with 5%, 20%, 35%, and 50% ACN or a sequence of these concentrations, the isolated peptides were desalted and analyzed *via* LC-MS/MS. Similar to the results obtained for the MA cell lines, we observed higher numbers of isolated MHC ligands for the HLA-A∗02:01 and HLA-B∗08:01 transfected cell lines with increasing concentrations of ACN (Fig. 7*A*). For COS-7 cells transfected with HLA-A∗03:01, a concentration of 20% ACN yielded the highest number of peptides with clear advantages of a sequential approach, as we observed for HLA-B∗08:01 as well (Fig. 7*A*). Notably, the relative increase in MHC ligands was comparable to the results obtained in our MA analyses (Fig. 7*B*). The differences in isolation efficiency aligned well with the peptides’ average level of hydrophobicity as determined for each condition through GRAVY scores (Fig. 7*C*).

In additional validation experiments, we used TAP-deficient T2 cells that were loaded externally with three HLA-A∗02-binding peptides (ALNEQIARL, ALNEKLVNL, and MLANDIARL) at a concentration of 1 μg/ml, which differed in their GRAVY score by approximately 0.3 points each. We then isolated peptides using our described method, limiting the ACN concentration to 5%, 35%, and 50%. Still, we added stable-isotope-labeled amino acids to estimate the number of presented epitopes per cell of the respective MHC ligands. Here, we made two interesting observations. First, the number of recovered epitopes per cell would range widely from just a few epitopes per cell in the 5% ACN condition to hundreds of molecules per cell in the 35% ACN condition to over 1000 molecules per cell for the ALNEQIARL peptide and over 10,000 molecules per cell for the ALNEKLVNL and MLANDIARL peptides in the highest ACN condition. Second, the ratio of recovered peptides was about 2-fold higher for ALNEKLVNL and MLANDIARL compared to ALNEQIARL in the 5% condition but almost 5-fold in the 35% condition and finally 10-fold higher in the 50% condition (Fig. 7*D*). All these results highlight not only that it needed 50% to (potentially) fully recover the tested HLA-A∗02:01 ligands but also that the relative measurement that might be used as a biological readout could vary strongly depending on the amount of ACN used for peptide isolation.

To finally transfer these findings to highly biologically relevant MHC ligands like public neoepitopes (mutated MHC ligands derived from essential oncogenes) we transfected COS-7 cells with HLA-B∗08:01 and an mRNA encoding the H1047L-mutated PIK3CA gene since this combination has been described to result in the presentation of the public neoepitope FMKQMNDAL (42). Furthermore, we assumed that this peptide sequence, given the two methionines and their potentially oxidized state, could be detected in four different variants, which we termed MM, mM, Mm, and mm (with M indicating a nonmodified methionine and m indicating an oxidized methionine) depending on which methionine is oxidized or not. After isolating MHC ligands with our five different ACN conditions, we analyzed the data with Skyline and combined the peak areas from all four potential precursors which demonstrated a constant increase in isolation efficiency throughout all conditions with a 7-fold difference between 5% ACN and the sequential isolation procedure (Fig. 7*E*). Even more interestingly, we observed a distinct distribution of detected oxidized or nonoxidized variants across all different conditions. This is illustrated by the nondetection of the mm and MM variants in the 5% condition as well as an absolute equal distribution between all four variants (mm, Mm, mM, and MM), in the sequential elution strategy (Fig. 7*F*, supplemental. Fig. S7).

Altogether, these results illustrate nicely how not only the MHC alleles per se, as shown through monoallelic cell lines, but also the characteristics of individual peptides influence the recovery of these peptides. Especially, the quantitative amounts of recovered peptides and their modification status can be strongly dependent on the chosen isolation strategy.

### Developing ARDisplay-I—An MHC Ligand Prediction Tool With Improved Accuracy

As our results are highly dependent on the accuracy of prediction algorithms, such as netMHCpan, used to predict which MHC allele a given peptide might bind to, we sought a complementary predictor tailored to eluted-ligand data and MA samples that complement our isolation strategy. Our goal was to achieve improved performance and fewer ambiguous assignments in realistic MA settings. Since it has been repeatedly shown that models based solely on BA measurements are insufficient for accurate detection of MHC ligands, ARDisplay-I is trained using MS-eluted positives and biologically informed decoys. Decoys were derived from nonpresented fragments of source proteins that uniquely match the observed ligands, then filtered to hard negatives; for training, we limited decoys to pHLA pairs with predicted BA < 2000 nM (MHCflurry (15)), thereby combining EL signal with a BA prior while maintaining challenging negatives. Such a holistic approach can lead to higher accuracy as the entire antigen processing and presentation pathway is taken into account implicitly (29). Currently, many computational models are trained to identify the motifs related to pMHC binding and presentation on the cell surface (14, 15, 16, 17, 18, 19). Even though BA predictors have been thoroughly tested (as they were available much earlier than EL predictors), they do not extend well to the task of EL prediction (17, 31).

Our primary evaluation uses an independent MS experiment generated in this study as a held-out test for ARDisplay-I. This test set was never used for model training, early stopping, threshold calibration, or hyperparameter selection for our model. However, because we do not have access to the training data of external tools, we cannot guarantee that some test peptides (especially widely reported ligands) are not present in their training corpora. To maintain comparability, we did not remove any peptides from the training set as doing so only for ARDisplay-I could bias the comparison. This choice may advantage the tools with the highest overlap, so we explicitly report this as a limitation.

To reliably compare the effectiveness of the different algorithms for EL and BA prediction, we computed model predictions (Supplemental Data 6) on a fixed, held-out test set composed exclusively of the MS data generated in the present study from three different cell lines (Nalm6, LP1, and JJN3) expressing, in total, 17 distinct MHC class I alleles. We obtained more than 32,000 HLA ligands presented on the cell surface. Moreover, because MS results do not directly provide information about peptides that exist within a cell but are not displayed on its surface *via* MHC molecules, we constructed artificial negative examples from the remaining (nonpresented) fragments of original proteins that match uniquely to the MHC ELs.

**Fig. 8:**
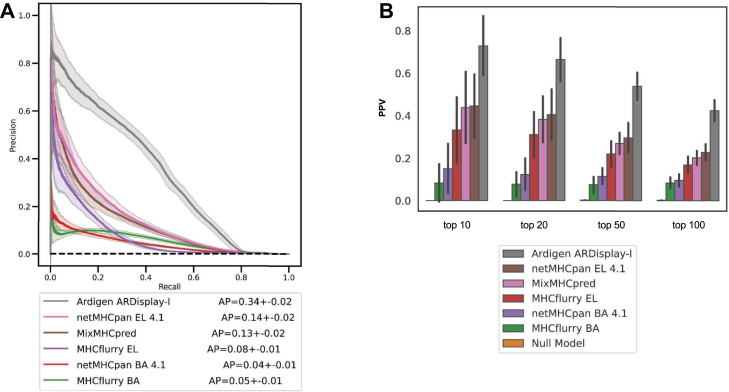
**Comparison of Ardigen ARDisplay-I performance with the performance of selected methods.***A*, precision-recall curve (PR curve) for the selected methods—see *Materials and Methods* sections for more details. Random resampling of observations from all three cell lines combined was performed 100 times to assess the variability of the metric, thereby estimating the average performance and its confidence band. For each iteration, a set of 500 positives and 500k negatives (×1000 number of positives) was randomly selected. AP stands for average precision and refers to the area under the precision-recall curve. *B,* precision (also PPV) for four thresholds, *i.e.,* top-10, 20, 50, & 100 MHC ligands selected by each method. The same sampling procedure has been applied as in (*A*). MHC, major histocompatibility complex.

Consistently with previous results (43), we showed that using BA predictors is not sufficient to accurately predict HLA ligands, and the performance of such algorithms is up to 3.5 times lower than that of EL predictors offered by the same groups (Fig. 8*A*). Moreover, on the same held-out MS dataset, ARDisplay-I achieves a significantly higher AP than the remaining methods trained on mass spec data—over 2.4 times higher than netMHCpan v4.1 (EL), over 2.6 times higher than MixMHCpred, and over 4.2 times higher than MHCflurry (EL). Despite these gains, our metrics remain far from theoretical optimum; there is a substantial gap to a perfect model (*i.e.,* the AP of 1), indicating that our benchmark approximates a more realistic and stringent scenario than common validation schemes typically proposed by other groups (14, 15, 44). This might be related to our approach to artificial decoy generation and/or a higher ratio of negative to positive observations in our test set (we use a ratio of 1000:1 negatives to positives, consistent with the average number of obtained *versus* combinatorially possible ligands per protein).

To analyze the performance of each method, we presented the precision (PPV) for the cases when the top-10, -20, −50, and −100 peptides are selected as HLA ligands (presented peptides) with each model (Fig. 8*B*, supplemental Table S1). Within the top-100 HLA ligands predicted by each algorithm, ARDisplay-I is shown to detect 42.7 ± 4.7 of the presented peptides. In contrast, netMHCpan v4.1 (EL) detects only 22.3 ± 3.5, MixMHCpred detects 20.8 ± 3.6, whereas MHCflurry (EL) detects only 17.7 ± 3.5, and the BA models detect around 10 and 8.5, respectively. Therefore, at this threshold (for top-100), ARDisplay-I detects 91% more ligands than netMHCpan, 105% more ligands than MixMHCpred, and 141% more ligands than MHCflurry.

Next, we compared the results of both prediction tools for our previous data with different ACN elution conditions. Although similar overall distributions were observed, ARDisplay-I consistently returned smaller quantities than netMHCpan (Fig. 1, *A*–*C*). The differences between the individual MHC alleles were profound; for example, ARDisplay-I predicted far fewer MHC ligands being presented by HLA-A∗01:01, A∗26:01, A∗33:01, B∗18:01, B∗35:03, C∗04:01, and C∗08:02, while for C∗07:01 as well as C∗07:02, the opposite was true (Fig. 1, *D*–*F*). Consequently, the overlap of predicted binders between the two algorithms was incomplete, and the total number of predicted MHC ligands was up to 24% higher (for LP-1 cells in Fig. 2*C*, 11470 HLA ligands compared to 9348 HLA ligands) in the netMHCpan group (Fig. 2, *A*–*C*, supplemental data S4). This divergence is consistent with our emphasis on hard-negative construction and stricter training regimes that appear to reduce false-positive51 inflation in certain allele contexts but may also lower recall in others as discussed in the next two paragraphs.

### Ardigen ARDisplay-I Identifies Specific MHC Alleles Responsible for Peptide Presentation More Accurately than State-Of-The-Art Tools

To assess whether the differences between the benchmarked methods are biologically meaningful, we performed a complementary analysis by cross-referencing against a collection of experimentally validated single-allelic pHLA data from multiple sources, including Sarkizova (27), Abelin (12), Di Marco (45), Faridi (46), Chen (25), Mei (47), and the IEDB database (48) (http://www.iedb.org/) (Figs. 2*D* and 3*D*). We compared the overlap and complementarity between three top-performing predictors [ARDisplay-I, netMHCpan (EL), and MixMHCpred], by first quantifying how many peptides each method calls positive and then asking what fraction of those calls are experimentally verified pHLA pairs.

### netMHCpan (EL) Overlap and Experimental Confirmation

When comparing ARDisplay-I with netMHCpan (EL) on both JJN3 and Nalm6 cell lines (Fig. 2, *A* and *B*), we see that they share more than 89.7% of predicted positives, with the majority of shared calls corresponding to known true pHLA pairs, aka, peptides experimentally confirmed as presented on the cell surface *via* specific MHC alleles. Specifically, this is over 72% for JJN3 and 63% for Nalm6 (Fig. 2*D*). The overlap not reaching 100% on any of the three cell lines (between 76–92%, Fig. 2, *A*–*C*) and the modest absolute number of confirmed pairs likely reflect the intrinsic ambiguity of multiallelic settings: many peptides observed by MS must be presented by at least one co-occurring allele, yet a matching single-allelic confirmation may not exist.

### MixMHCpred Overlap and Experimental Confirmation

When comparing ARDisplay-I with MixMHCpred on JJN3 and Nalm6, the two methods share 89.2% and 89.4% of predicted positives, respectively (Fig. 3, *A* and *B*, n = 6307 and 7372). Among these shared calls, 72.4% (JJN3) and 63.7% (Nalm6) are supported by single-allelic reference data (Fig. 3*D*), indicating that a substantial fraction of the intersection corresponds to experimentally verified pHLA pairs. As with netMHCpan, incomplete coverage of monoallelic assays likely limits the absolute number of confirmed assignments.

In LP1, the overlap between ARDisplay-I and MixMHCpred is again lower (74.6% shared positives, Fig. 3*C*), and only 39.4% of these are known true positives (Fig. 3*D*). This mirrors the cell line-specific behavior observed with netMHCpan, underscoring that the origin of training data and tissue context can highly influence model generalization.

MixMHCpred produces 2.7 to 8.0 × more unique positives than ARDisplay-I across the analyzed cell lines (Fig. 3, *A*–*C*) with threshold selected so that the number of overlapping peptides is similar to the previous comparison with netMHCpan (Fig. 3). However, only 1.63 to 8.42% of these unique calls are supported by monoallelic datasets (Fig. 3*D*). In contrast, 2.93 to 34.12% of ARDisplay-I’s unique predictions are experimentally confirmed, yielding a 1.8 to 7.17 × higher fraction of uniquely identified, allele-matched ligands for ARDisplay-I relative to MixMHCpred (Fig. 3*D*).

These results indicate that ARDisplay-I not only matches but often exceeds state-of-the-art performance where single-allele ambiguity is highest: its unique predictions are far more likely to replicate in independent single-allele datasets, whereas many of the additional unique calls from netMHCpan (EL) or mixMHCpred remain unconfirmed. While incomplete single-allele coverage limits absolute confirmation rates, the enrichment for experimentally validated pairs among ARDisplay-I’s unique calls supports improved biological specificity in assigning peptides to their presenting alleles.

### Additional Validation on Post-Training Datasets (Stricter Benchmark)

While the biological utility of ARDisplay-I is evident, the benchmark described above suffers from the potential overlap between the training data of the compared models and the pHLA pairs (the cross-referenced monoallelic datasets). To address this concern and provide a stricter validation approach, we propose an alternative, more rigorous evaluation method that focuses exclusively on pHLA pairs derived from IEDB and experiments conducted in 2022 and later, *i.e*., following the training of both ARDisplay and netMHCpan. As expected, this approach significantly reduced the number of available cross-reference data points, as we deliberately excluded most of the previously confirmed MHC ligands.

Under this post-training benchmark, ARDisplay-I again outperforms netMHCpan (EL) and mixMHCpred in attributing peptides to their presenting alleles (Figs. 2*E* and 3*E*). As expected with fewer references, confirmation rates decreased for all three methods, but still, ARDisplay-I achieved 6.85 to 19.95× higher fractions of *uniquely identified, experimentally confirmed, allele-matched* ligands than netMHCpan (Fig. 2*E*) and 1.38 to 2.57× for mixMHCpred (Fig. 3*E*).

These results indicate better generalization to unseen data and reinforce the biological specificity of ARDisplay-I under a more rigorous, post-training validation regime.

## Discussion

The isolation and definition of MHC ligands, which might serve as targets for T cell-inspired therapies, can already be biased during the isolation process of MHC-bound peptides. As we and others have demonstrated before, the hydrophobicity of peptides is an important factor that must be taken into account (5, 6, 13). Here, we broaden the knowledge about specific MHC alleles that benefit from more rigorous isolation strategies and demonstrate that PTMs also contribute to the detection or nondetection of MHC ligands. Still, although it has become evident that specific MHC alleles benefit from a more stringent use of ACN during isolation, it is also clear that within the group of peptides isolated from 1 cell line or MHC, allele-specific subgroups will always be favored by distinct isolation procedures which also include other steps of the isolation process like the use of mild acid elution compared to MHC immunoaffinity isolation (5). Thus, it may be ideal to separate MHC ligands and higher molecular parts of the MHC complexes using RP-HPLC, as demonstrated earlier (49). However, as this isolation is much more time-consuming and requires additional mass spectrometry runs per sample, our method may provide a more effective strategy. On a single MHC ligand level, it might be worth determining the ideal elution conditions, as the use of too high or too low ACN concentrations might lead to insufficient isolation of the investigated MHC ligands, which, especially in quantitative immunopeptidomics, could lead to overestimation or underestimation of the presented MHC ligands. A parameter that becomes increasingly important for the design of TCR- and TCR mimic-based immunotherapies (48, 50). We also highlighted how different oxidized variants of a PIK3CA-derived neoepitope are recovered under varying isolation conditions. This has important implications for such low-abundance targets, as the most effective elution strategy should be chosen to ensure the detectability of a specific target. However, a general recommendation on the ACN concentration based on the HLA alleles present in a sample cannot be given. While our data suggest that A∗02 containing samples might benefit the most from including higher concentrations of ACN (e.g. 50%), the choice of ACN concentration should be based on the biological question. If a broad range of HLA ligands should be recovered, a sequential elution strategy might be most beneficial, whereas for specific epitope detection, an estimate based on previous retention times might inform the choice of ACN concentration.

Alongside these biochemical improvements, we developed ARDisplay-I, a custom HLA ligand prediction model, and compared its predictive performance with other commonly used methods. We utilized the dataset generated in the present study, which enables an unbiased comparison, as the dataset was not used in training any of the benchmarked models. We found that ARDisplay-I achieves 2.4 times higher AP than netMHCpan 4.1 (EL), over 2.6 times higher than MixMHCpred, and 4.2 times higher AP than MHCflurry (EL). Consistent with prior observations, using BA predictors proves to be insufficient for the accurate prediction of HLA ligands. Notably, all our metrics remain far from the theoretical optimum, suggesting that the benchmark explores a more realistic and stringent scenario than the validation schemes typically used; hence, it better reflects the biological situation to which these computational models are applied. Although fewer peptides were assigned to HLA alleles compared to netMHCpan, we assume that this is the result of a reduction in the fraction of false positives, as these prediction tools still tend to overestimate the number of binders to the respective alleles (51). This assumption is supported when cross-referencing the data with MS readouts from monoallelic cell lines.

Overall, our study provides the field of immunopeptidomics with two important advancements. First, we define MHC alleles that benefit the most from stringent separation conditions for peptides and MHC complexes when loaded onto C18 columns, which helps to identify ideal isolation conditions for specific MHC ligands. Second, we developed a new prediction tool, ARDisplay-I, that offers improved accuracy in predicting HLA ligands and assigning them to HLA alleles, outperforming other state-of-the-art algorithms.

## Data availability

The benchmark dataset used in this study includes data from myeloma and leukemia cell lines (JJN3, LP-1, and Nalm-6), with MS-generated results covering over 32,000 HLA ligands. This dataset will be made publicly available upon publication, ensuring reproducibility and accessibility.

The benchmark dataset has been deposited at the PRIDE archive. Submission details:

Project Name: Identification of MHC ligands through allele-guided isolation combined with machine learning for specific MHC assignment

Project accession: PXD061101 Project DOI: 10.6019/PXD061101

For support or inquiries, feel free to contact ardisplay@ardigen.com.

## Supplemental data

This article contains supplemental data (12, 14, 15, 18, 27).

## Conflicts of interest

MGK is a consultant to Ardigen, Biocopy, and T-knife. Authors P. S., P. B., B. K. J., M. J., V. M. P., A. S. D., R. S., J. K., and A. B. were employed by Ardigen SA. The remaining authors declare that the research was conducted in the absence of any commercial or financial relationships that could be construed as a potential conflict of interest.

## Supplementary data

Supplementary Figure S1 **Global HLA allele frequency of HLA alleles used in the investigated cell lines.***A*, absolute individual frequencies of the HLA alleles present in JJN3, LP-1, and Nalm-6 cells. *B*, cumulative frequencies of investigated HLA alleles worldwide.

Supplementary Figure S2 **Relative isolation efficiency of modified MHC ligands.** Isolation efficiency of different modified MHC ligands in JJN3, LP-1, and Nalm-6 cells. Results from four biological replicates and three different cell lines were pooled. The number of isolated modified MHC ligands was normalized to the 20% ACN condition. The following modifications were tested: (*A*) oxidation, (*B*) methylation, (*C*) dimethylation, and (*D*) acetylation with the cumulative frequencies of investigated HLA alleles worldwide. Multiple comparisons were done using a Friedman test.

Supplementary Figure S3 **Overlap of modified MHC ligands in various ACN elution conditions.** Shared modified MHC ligands across different ACN conditions were analyzed via five group venn diagrams. The following modifications are shown: (*A*) methylation, (*B*) dimethylation, (*C*) oxidation, (*D*) acetylation, and (*E*) phosphorylation.

Supplementary Figure S4 **Overview of statistical difference in MHC ligand isolation yields between MHC alleles using various ACN conditions.** Heatmap depicting p-values for multiple comparisons between investigated MHC alleles. The relative increase of MHC ligands isolated compared to the overall rise in MHC ligands was the basis for the initial statistical comparison. Two-way ANOVA was used for multiple comparisons.

Supplementary Figure S5 **Reanalysis of overall and MHC allele-specific MHC ligand using PEAKS.***A*, unique MHC ligands isolated from the same pool of peptide:MHC using various concentrations of acetonitrile (ACN) in JJN3, LP-1, and Nalm-6 cells. *B*, relative changes for the yields of unique MHC ligands between different ACN elution conditions in JJN3, LP-1, and Nalm-6 cells. *C*-*E*, absolute numbers of MHC ligands assigned to a specific MHC allele. All experiments were performed in biological quadruplicates.

Supplementary Figure S6 **Relative isolation efficiency of modified MHC ligands using PEAKS.** Isolation efficiency of different modified MHC ligands in JJN3, LP-1, and Nalm-6 cells. Results from four biological replicates and three different cell lines were pooled. The number of isolated modified MHC ligands was normalized to the 20% ACN condition. The following modifications were tested: (*A*) oxidation, (*B*) cysteinylation, and (*C*) phosphorylation. Multiple comparisons were done using a Friedman test.

Supplementary Figure S7 **Peak areas of the public neoepitope FMKQMNDAL in cotransfected COS-7 cells.** Peak areas from the public neoepitope FMKQMNDAHL presented on COS-7 cells cotransfected with B∗08:01 and PIK3CA H1047L. The neoepitope presentation was assessed in four different states depending on the oxidation state of the two methionines present (M=nonoxidized methionine and m=oxidized methionine) in the neoepitope, called (*A*) MM (FMKQMNDAHL), (*B*) mM (FmKQMNDAHL), (*C*) mM (FMKQmNDAHL), and (*D*) mm (FmKQmNDAHL). Suppl. Table 1. Benchmark PPV values supporting Fig. S5*B*. It contains mean PPV (positive predicted value) and standard deviation computed from 100 resampling experiments on subsets of the benchmark dataset.

Supplementary Data 6 **Predictions from consecutive models and their versions.** Side-by-side predictions (probabilities or binding affinity nanomoles [nM]) produced by consecutive models, enabling traceability of changes across versions. Columns (per entry): peptide, set of HLA allele associated with a given cell line, predictions from MixMHCpred, MHCflurry BA/EL, netMHCpan BA/EL 4.1, and ARDisplay-I, and information of presentation status (“presented”).

Supplementary Data 1 **Unique HLA ligand identifications for the cell lines LP-1, Nalm-6, and JJN3.** These spreadsheets contain all HLA ligand identification from four replicates and different ACN concentrations from cell line LP-1.

Supplementary Data 2 **Unique HLA ligand identifications for the cell lines LP-1, Nalm-6, and JJN3.** These spreadsheets contain all HLA ligand identification from four replicates and different ACN concentrations from cell line Nalm-6.

Supplementary Data 3 **Unique HLA ligand identifications for the cell lines LP-1, Nalm-6, and JJN3.** These spreadsheets contain all HLA ligand identification from four replicates and different ACN concentrations from cell line JJN3.

Supplementary Data 4 **Predicted positive peptides supporting Figs. 7A-C and 8A-C.** This spreadsheet contains three separate sheets, one per cell line. The overlap column lists peptides predicted as presented/positive by all three methods, whereas Ardigen_ARDisplay (>=50%), netMHCpan (<2.0 %rank), and MixMHCpred (80% quantile) list peptides uniquely predicted by the respective method.

Supplementary Data 5 **Confirmed (TP, true positive) peptides supporting tables Figs. 7D-E and 8D-E.** This spreadsheet contains three separate sheets, one per cell line. It lists predicted positive peptides cross-referenced against experimentally validated single-allelic pHLA datasets. It contains two columns for different evidence source, match to (D) all mono-allelic datasets used broadly in the literature (“mono-allelic datasets (D)”) and (E) a stricter test matching to entries published after ARDisplay and netMHCpan training (“post-training datasets (E)”).

Supplementary Data 7 **Numeric values used for generating**Fig. 1, Fig. 2, Fig. 3, Fig. 4**, and**6**as well as**Supplemental Figs. S2**, and**S4–S6**.** This spreadsheet contains numeric values for all Figures and Supplemental Figures listed above.
