# Supplementary material for: Identification of MHC Ligands Through Allele-Guided Isolation Combined With Machine Learning for Improved MHC Assignment Using ARDisplay-I
Source: Mol Cell Proteomics. 2026 Mar 27;25(5):101560. doi: 10.1016/j.mcpro.2026.101560 (PMC13156753; doi:10.1016/j.mcpro.2026.101560)
Supplement: Supplementary Figure S1 — Global HLA allele frequency of HLA alleles used in the investigated cell lines.A, absolute individual frequencies of the HLA alleles present in JJN3, LP-1, and Nalm-6 cells. B, cumulative frequencies of investigated HLA alleles worldwide. [file mmc1.pdf]

Suppl. Figure 1

A

Coverage Class I	HLA-A*01:01 (9.08)	HLA-A*02:01 (21.95)	HLA-A*03:01 (8.79)	HLA-A*26:01 (2.95)	HLA-A*30:01 (1.96)	HLA-A*33:01 (0.89)	HLA-B*07:02 (6.61)	HLA-B*08:01 (5.42)	HLA-B*14:02 (1.45)	HLA-B*15:01 (4.32)	HLA-B*18:01 (3.73)	HLA-B*35:03 (1.70)	HLA-C*04:01 (10.57)	HLA-C*07:01 (10.25)	HLA-C*07:02 (11.38)	HLA-C*08:02 (2.14)	Total HLA hits
17.34%	+	-	-	-	-	-	-	-	-	-	-	-	-	-	-	-	1
39.08%	-	+	-	-	-	-	-	-	-	-	-	-	-	-	-	-	1
16.81%	-	-	+	-	-	-	-	-	-	-	-	-	-	-	-	-	1
5.82%	-	-	-	+	-	-	-	-	-	-	-	-	-	-	-	-	1
3.89%	-	-	-	-	+	-	-	-	-	-	-	-	-	-	-	-	1
1.77%	-	-	-	-	-	+	-	-	-	-	-	-	-	-	-	-	1
12.78%	-	-	-	-	-	-	+	-	-	-	-	-	-	-	-	-	1
10.55%	-	-	-	-	-	-	-	+	-	-	-	-	-	-	-	-	1
2.88%	-	-	-	-	-	-	-	-	+	-	-	-	-	-	-	-	1
8.44%	-	-	-	-	-	-	-	-	-	+	-	-	-	-	-	-	1
7.32%	-	-	-	-	-	-	-	-	-	-	+	-	-	-	-	-	1
3.36%	-	-	-	-	-	-	-	-	-	-	-	+	-	-	-	-	1
20.02%	-	-	-	-	-	-	-	-	-	-	-	-	+	-	-	-	1
19.44%	-	-	-	-	-	-	-	-	-	-	-	-	-	+	-	-	1
21.47%	-	-	-	-	-	-	-	-	-	-	-	-	-	-	+	-	1
4.24%	-	-	-	-	-	-	-	-	-	-	-	-	-	-	-	+	1
92.48%	1	1	1	1	1	1	1	1	1	1	1	1	1	1	1	1	16

Table header: HLA allele (genotypic frequency (%))

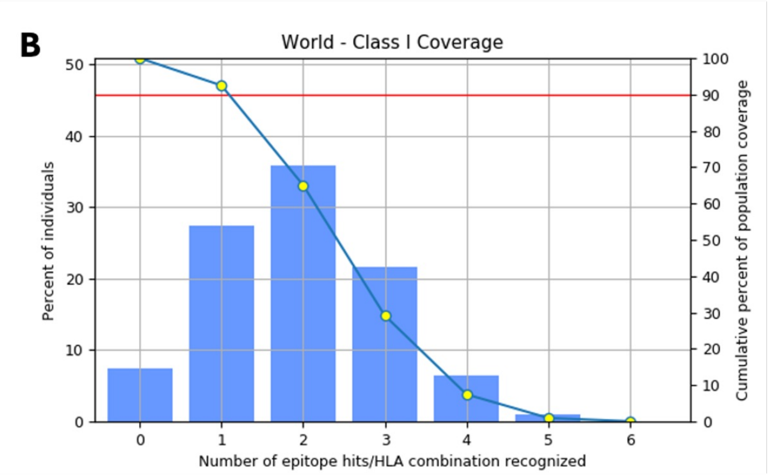

+ : restricted
- : not restricted
