# Supplementary figures and images for: Identification of MHC Ligands Through Allele-Guided Isolation Combined With Machine Learning for Improved MHC Assignment Using ARDisplay-I
Source: Mol Cell Proteomics. 2026 Mar 27;25(5):101560. doi: 10.1016/j.mcpro.2026.101560 (PMC13156753; doi:10.1016/j.mcpro.2026.101560)

Suppl. Figure 2

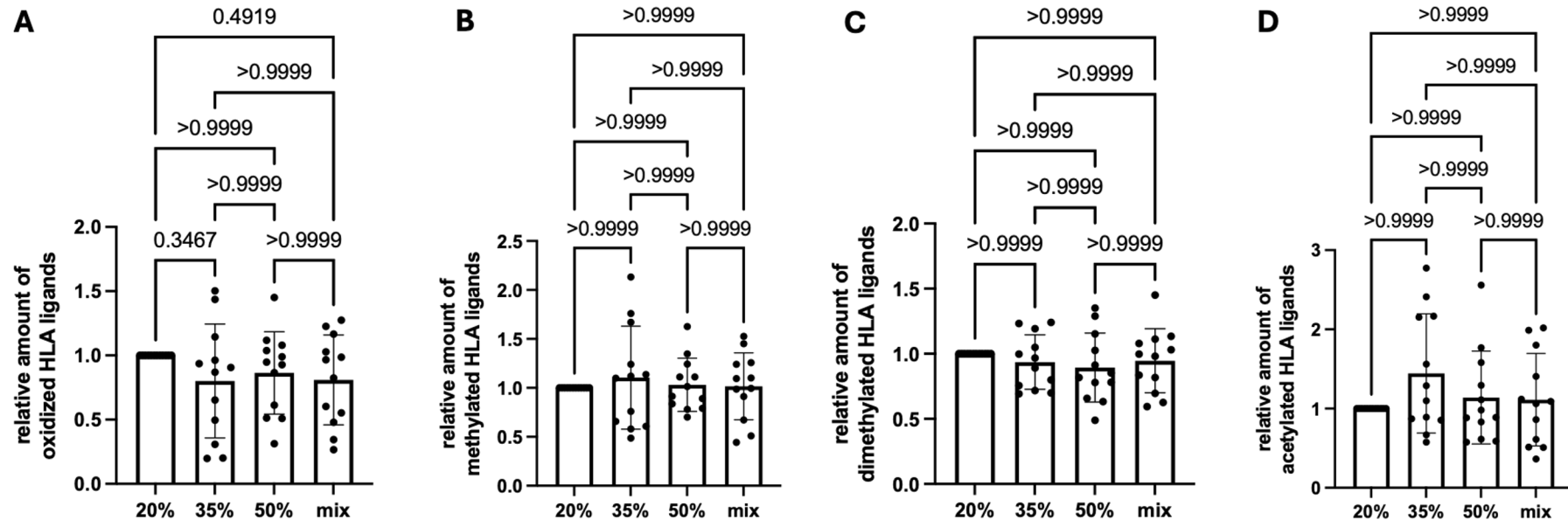

Supplement: Supplementary Figure S2 — Relative isolation efficiency of modified MHC ligands. Isolation efficiency of different modified MHC ligands in JJN3, LP-1, and Nalm-6 cells. Results from four biological replicates and three different cell lines were pooled. The number of isolated modified MHC ligands was normalized to the 20% ACN condition. The following modifications were tested: (A) oxidation, (B) methylation, (C) dimethylation, and (D) acetylation with the cumulative frequencies of investigated HLA alleles worldwide. Multiple comparisons were done using a Friedman test. [file mmc2.pdf]

Suppl. Figure 3

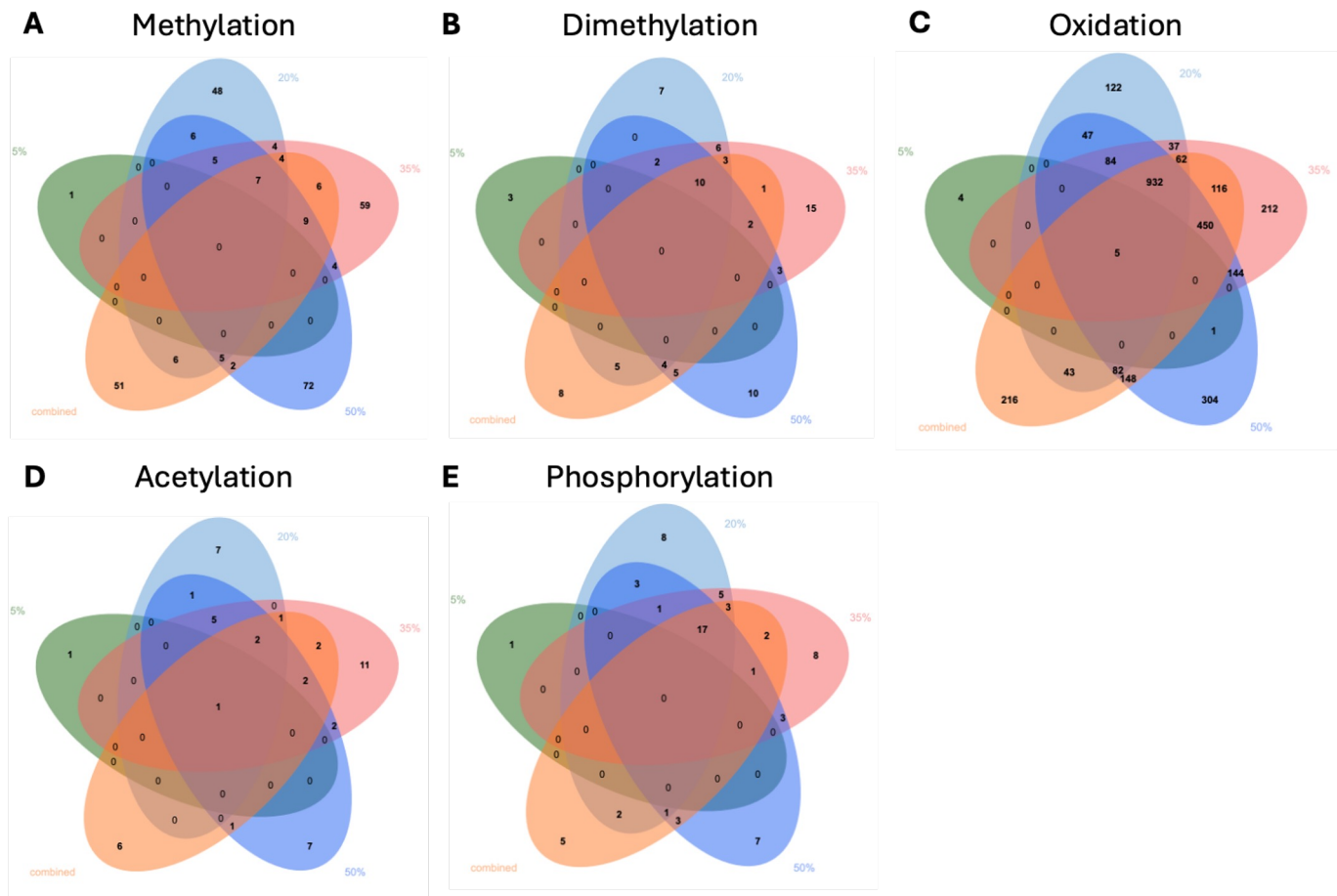

Supplement: Supplementary Figure S3 — Overlap of modified MHC ligands in various ACN elution conditions. Shared modified MHC ligands across different ACN conditions were analyzed via five group venn diagrams. The following modifications are shown: (A) methylation, (B) dimethylation, (C) oxidation, (D) acetylation, and (E) phosphorylation. [file mmc3.pdf]

Suppl. Figure 5

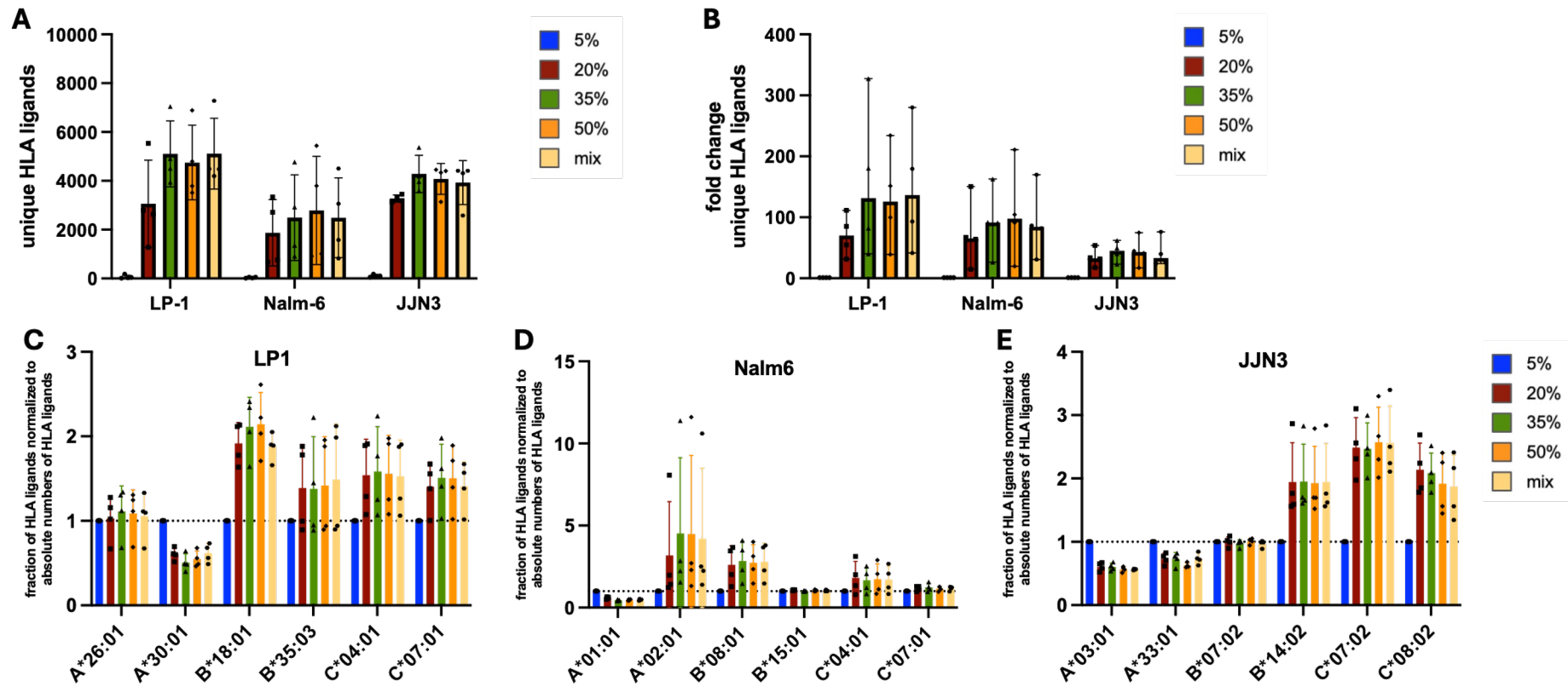

Supplement: Supplementary Figure S5 — Reanalysis of overall and MHC allele-specific MHC ligand using PEAKS.A, unique MHC ligands isolated from the same pool of peptide:MHC using various concentrations of acetonitrile (ACN) in JJN3, LP-1, and Nalm-6 cells. B, relative changes for the yields of unique MHC ligands between different ACN elution conditions in JJN3, LP-1, and Nalm-6 cells. C-E, absolute numbers of MHC ligands assigned to a specific MHC allele. All experiments were performed in biological quadruplicates. [file mmc5.pdf]

Suppl. Figure 6

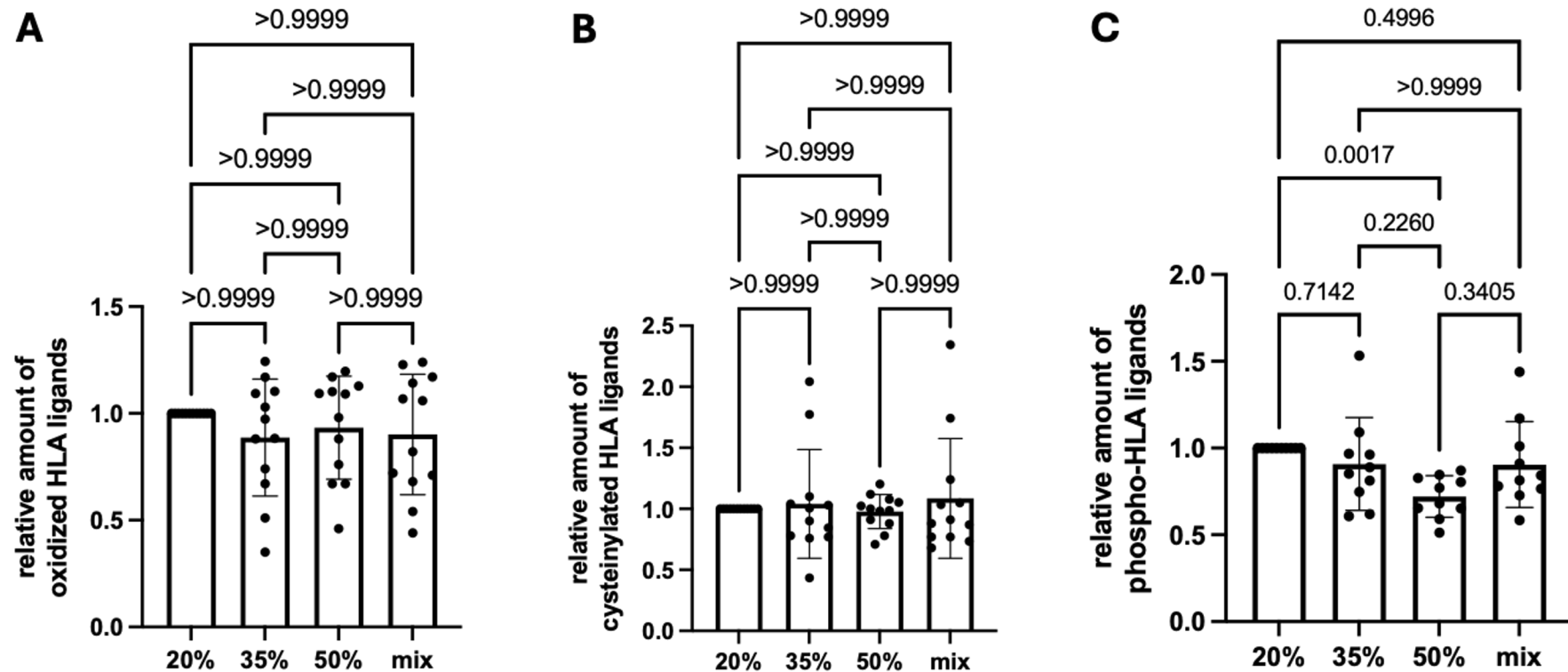

Supplement: Supplementary Figure S6 — Relative isolation efficiency of modified MHC ligands using PEAKS. Isolation efficiency of different modified MHC ligands in JJN3, LP-1, and Nalm-6 cells. Results from four biological replicates and three different cell lines were pooled. The number of isolated modified MHC ligands was normalized to the 20% ACN condition. The following modifications were tested: (A) oxidation, (B) cysteinylation, and (C) phosphorylation. Multiple comparisons were done using a Friedman test. [file mmc6.pdf]

# Suppl. Figure 7

**A**

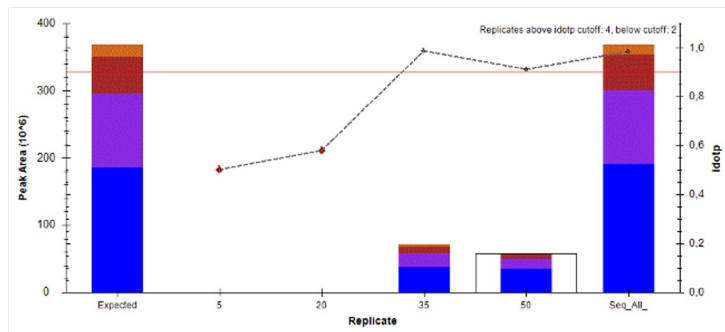

FMKQMNDAL (MM)

**B**

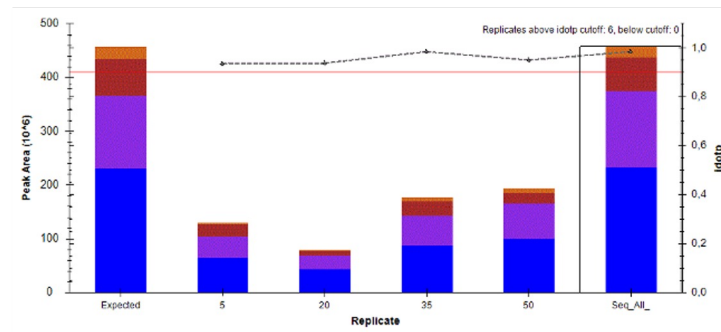

Fm[+15,995]KQMNDAL (mM)

**C**

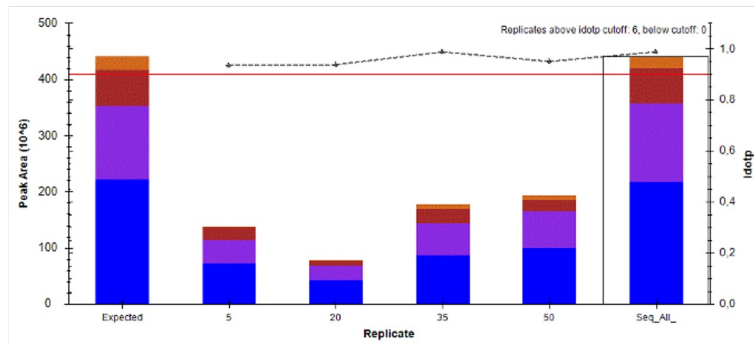

FMKQm[+15,995]NDAL (Mm)

**D**

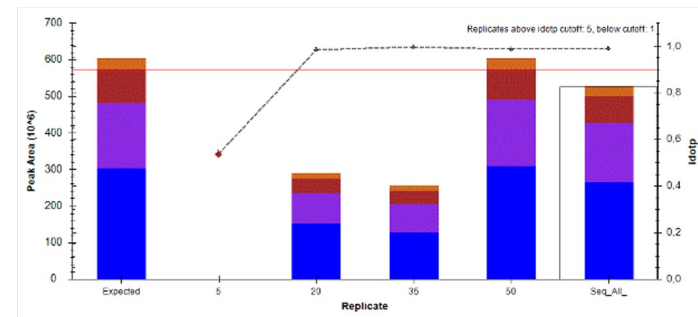

Fm[+15,995]KQm[+15,995]NDAL (mm)

Supplement: Supplementary Figure S7 — Peak areas of the public neoepitope FMKQMNDAL in cotransfected COS-7 cells. Peak areas from the public neoepitope FMKQMNDAHL presented on COS-7 cells cotransfected with B∗08:01 and PIK3CA H1047L. The neoepitope presentation was assessed in four different states depending on the oxidation state of the two methionines present (M=nonoxidized methionine and m=oxidized methionine) in the neoepitope, called (A) MM (FMKQMNDAHL), (B) mM (FmKQMNDAHL), (C) mM (FMKQmNDAHL), and (D) mm (FmKQmNDAHL). Suppl. Table 1. Benchmark PPV values supporting Fig. S5B. It contains mean PPV (positive predicted value) and standard deviation computed from 100 resampling experiments on subsets of the benchmark dataset. [file mmc7.pdf]
