# Supplementary material for: Identification of MHC Ligands Through Allele-Guided Isolation Combined With Machine Learning for Improved MHC Assignment Using ARDisplay-I
Source: Mol Cell Proteomics. 2026 Mar 27;25(5):101560. doi: 10.1016/j.mcpro.2026.101560 (PMC13156753; doi:10.1016/j.mcpro.2026.101560)

Suppl. Figure 4

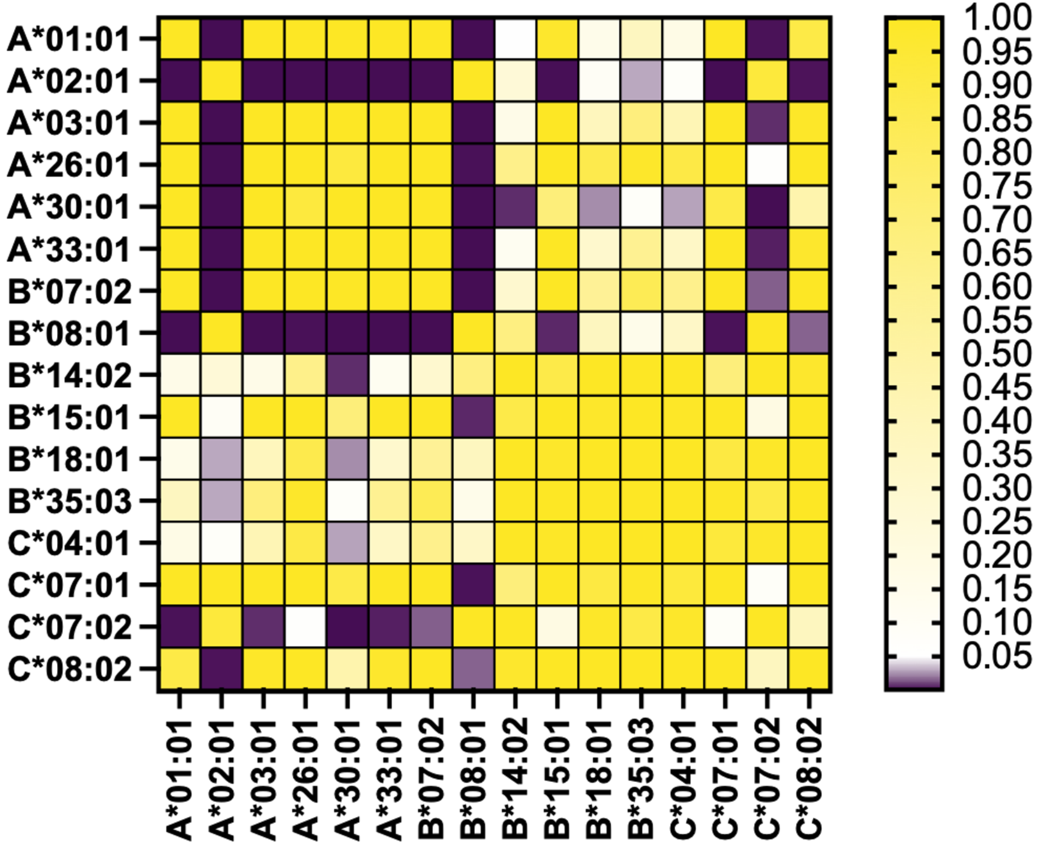

Supplement: Supplementary Figure S4 — Overview of statistical difference in MHC ligand isolation yields between MHC alleles using various ACN conditions. Heatmap depicting p-values for multiple comparisons between investigated MHC alleles. The relative increase of MHC ligands isolated compared to the overall rise in MHC ligands was the basis for the initial statistical comparison. Two-way ANOVA was used for multiple comparisons. [file mmc4.pdf]
